# Improved likelihood ratios for face recognition in surveillance video by multimodal feature pairing

**DOI:** 10.1016/j.fsisyn.2024.100458

**Published:** 2024-02-29

**Authors:** Andrea Macarulla Rodriguez, Zeno Geradts, Marcel Worring, Luis Unzueta

**Affiliations:** a Netherlands Forensic Institute, Laan van Ypenburg 6, The Hague, 2497GB, the Netherlands; b University of Amsterdam, Science Park 904, Amsterdam, 1098XH, the Netherlands; c Fundacion Vicomtech, Basque Research and Technology Alliance (BRTA), Mikeletegi Pasealekua 57, Donostia-San Sebastian, 20009, Spain

**Keywords:** Face recognition, Video processing, Face image quality, Likelihood ratio, Multi-modal analysis, Super resolution

## Abstract

In forensic and security scenarios, accurate facial recognition in surveillance videos, often challenged by variations in pose, illumination, and expression, is essential. Traditional manual comparison methods lack standardization, revealing a critical gap in evidence reliability. We propose an enhanced images-to-video recognition approach, pairing facial images with attributes like pose and quality. Utilizing datasets such as ENFSI 2015, SCFace, XQLFW, ChokePoint, and ForenFace, we assess evidence strength using calibration methods for likelihood ratio estimation. Three models—ArcFace, FaceNet, and QMagFace—undergo validation, with the log-likelihood ratio cost (*C*_*llr*_) as a key metric. Results indicate that prioritizing high-quality frames and aligning attributes with reference images optimizes recognition, yielding similar *C*_*llr*_ values to the top 25% best frames approach. A combined embedding weighted by frame quality emerges as the second-best method. Upon preprocessing facial images with the super resolution CodeFormer, it unexpectedly increased *C*_*llr*_, undermining evidence reliability, advising against its use in such forensic applications.

## Highlights

• Study tackles the problem of accurate likelihood ratios in surveillance video face recognition.
• Proposes a frame selection method to enhance forensic face recognition by pairing frames based on quality and face attributes.
• Optimal frame selection framework validated across various models and datasets.
• As additional result, the study reveals that the use of super-resolution preprocessing via CodeFormer adversely affects the reliability of forensic face recognition.

## 1 Introduction

Automated Face recognition (FR) is a method that has become increasingly important in recent years, particularly in the field of forensic investigation [1]. With the proliferation of surveillance cameras and the capture of images of criminal events, the comparison of faces has become a key tool for gathering intelligence, guiding investigations, and providing evidence in court [1,2]. While deep-learning based FR methods have demonstrated strong recognition performance for still images [3], such as those in the Labeled Faces in the Wild (LFW) dataset [4], video-based FR has not been as widely developed by the research community [5]. Video FR, however, offers additional information, such as temporal details and multiple views on the same person, which can be used in conjunction with frame based face recognition techniques to quickly identify subjects of interest in CCTV footage [6].

**Fig. 1:**
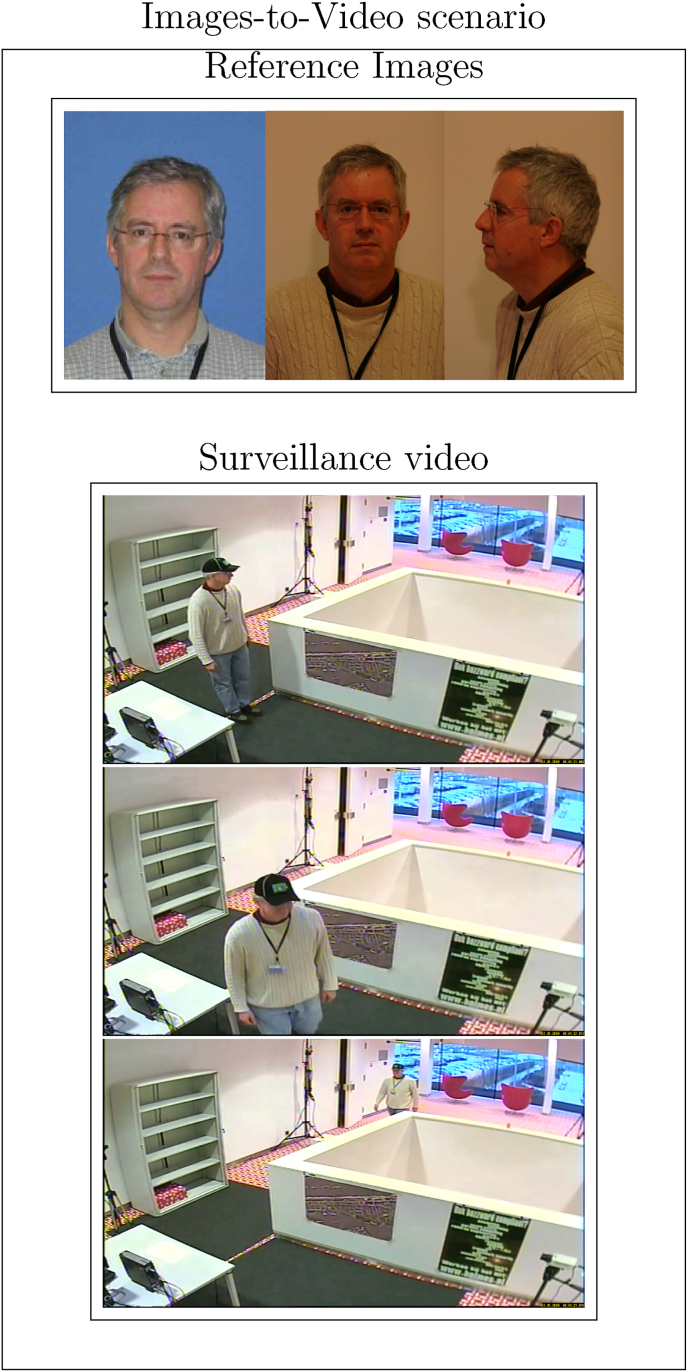
Example of images-to-video scenario. Images taken from Ref. [13]. The reduced quality in this case is primarily attributed to the following factors: the challenging pose of the face, the subject wearing a cap, and increased subject distance.

Despite the potential benefits of video-based FR, the process of analyzing such a large amount of data for each video is challenging due to the time needed to deal with all frames. Not all the frames in the video might be of equal importance though. Some frames can be useless for recognition due to low video quality, motion blur, occlusions, and frequent changes in the scene [7,8] (see Fig. 1 for examples). An obvious method would then be to measure such characteristics and discard frames of low quality. Some works focusing on face image quality (FIQ) [[9], [10], [11]], however, have indicated that using human-based attributes for face image quality assessment might not be ideal. Aspects that humans perceive as affecting the quality of an image, such as illumination or pose, may not be the best characteristics for the face recognition system being used. The references above use the SER-FIQ, MagFace, and SDD-FIQA face image quality assessment in deep learning based methods to test on IJB-C [12] videos, and show that for 1:1 recognition on individual frames these assessment methods yield significant improvements. In current systems, image quality measures incorporating spatio-temporal information are not used.

So how to evaluate which method is best in images-to-video scenarios? In automated facial recognition systems, the similarity between two samples is usually reported in one or several score values intrinsic to each version of the facial recognition algorithm used [3]. To allow comparisons between facial scores from different face recognition systems, as well as for such an automated comparison to be useful within a framework that assesses and measures effectiveness (‘evaluative forensic framework’), there is a need to map the output scores to a Likelihood Ratio (LR) [14]. An ‘evaluative forensic framework’ refers to a systematic approach used for assessing and quantifying the performance, reliability, and validity of forensic methods. LR is defined as the probability of the evidence given hypothesis *H*_0_ i.e., the probability of the reference being the same person as in the video, divided by the probability of the evidence given the alternative hypothesis *H*_1_ i.e., the probability of the reference being a different person than the one appearing in the video. A possible approach to achieve this is the use of a score-to-LR mapping as a post-processing step in an existing score-producing facial recognition system [15]. Once a model for score-to-LR mapping has been set up, the forensic reporting can be presented using a level of conclusion, where each grade on the scale is connected to an interval of LR values [2,16].

In this paper, which is an extension of our conference paper [17], we propose a novel method for images-to-video face recognition in realistic forensic scenarios. We leverage a model that pairs face images based on multimodal face feature data, such as face attribute characteristics and FIQ. The aim is to accurately estimate likelihood ratios (LRs) for face recognition systems in practical settings. Our particular focus is on scenarios where multiple reference images of a suspect are available, and to verify if this person is the same individual appearing in a surveillance video. Previous studies, such as the work of Molder et al. [15], Rodriguez et al. [18], and Jacquet et al. [2], have explored LRs in face recognition in still images, employing different techniques and considering various scenarios. Despite these contributions, there are still open questions, particularly regarding the accuracy of estimating LRs and their effective application in a forensic context. This paper aims to address these gaps by improving the accuracy of LR estimation in automated face recognition using image-to-video comparisons, building on the work of researchers like Zheng et al. [8] and Huo et al. [19]. We apply three calibration methods (random, attribute-based and quality-based) to estimate LRs and validate the results using the log-likelihood ratio cost (*C*_*llr*_). Our contributions include the following:

1) **MultiModal Feature Pairing** using FIQ to select frames with the highest quality and highest number of common attributes (soft labels), and combining them through a weighted average.
2) **Calibration** involving selection of random pairs with the same attributes and same FIQ as the test pairs.
3) **Validation** of the LR estimation system against a forensic test performed with human experts.
4) **Preprocessing with Super Resolution** method CodeFormer for preprocessing facial images and evaluate its effect on the *C*_*llr*_.

**Fig. 2:**
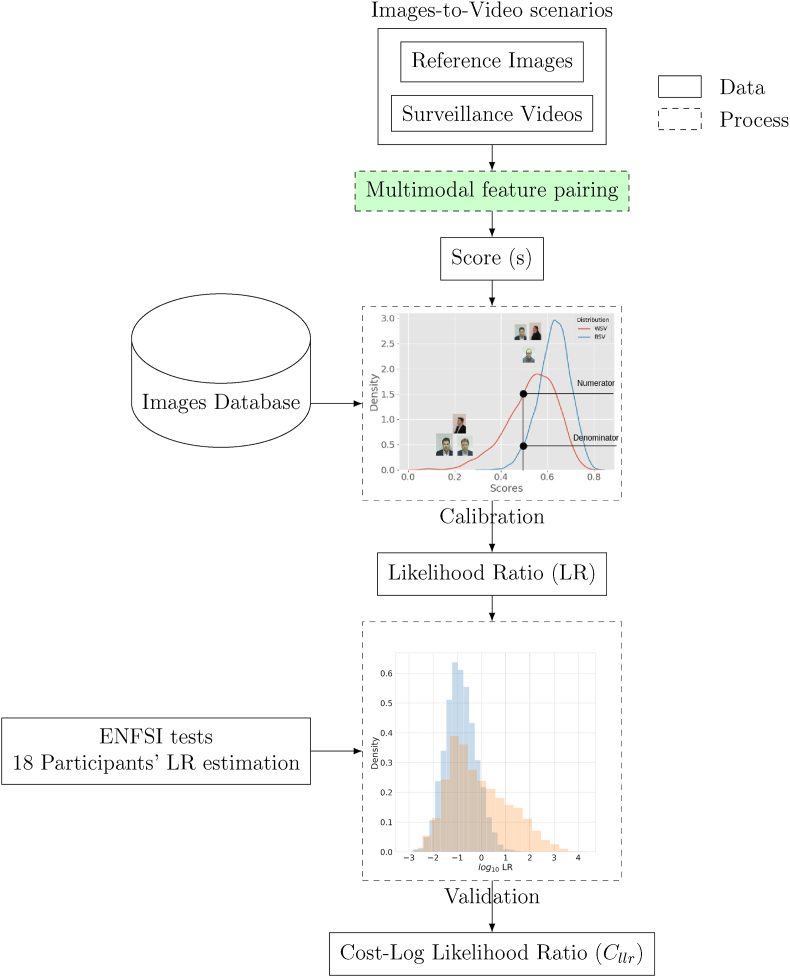
Workflow of the LR computation and validation process in ENFSI 2015 proficiency test [23].

The current study begins by providing an overview of the relevant literature pertaining to the estimation of likelihood ratios, face recognition in video, and the incorporation of FIQ in face recognition in images-to-video scenarios. Following this, the methodology for pairing and calibration is presented. The experiments and associated results are then discussed. The paper concludes with a discussion of the findings and implications. The workflow for the computation of the Likelihood Ratio, giving a blueprint for the paper, is depicted in Fig. 2.

Compared to our earlier conference paper [17], this study introduces several significant advancements. Firstly, we implement a new computational model, CodeFormer [20], designed to optimize face recognition performance. Secondly, we expand our dataset collection to include XQLFW [21] and ChokePoint [22], enriching the empirical foundation of our research. We offer enhanced interpretability of our results by introducing a sunburst diagram as a novel visualization tool to better understand the relationships between various facial attributes and image quality. Finally, the text has been significantly extended to give more insight in the methodologies and results.

## 2 Related work

Likelihood ratios (LRs) have been applied in the field of face recognition in different ways. Molder et al. [15] test score-to-LR models in forensic data and find that the performance of the models is highly dependent on the available training data. Rodriguez et al. [18] and Jacquet et al. [2] also focus on this topic, with the former using facial attributes and quality scores to improve LR estimation, and find that current commercial software outperforms open-source software. The latter reference explores the importance of LR in face recognition and assesses the performance of the model with respect to its discriminating power and calibration state. While these studies have made significant strides, the current state of LR research in video-based face recognition remains incomplete. An open question is how to accurately estimate LRs for face recognition systems in practice and how they can be effectively deployed in a forensic context, particularly when analyzing video sequences rather than isolated frames.

Spatio-temporal face recognition in videos has also been a topic of research. Zheng et al. [8] propose a system for image-to-video face recognition in unconstrained conditions, composed of modules for landmark detection, face association, and face recognition. They perform experiments on video datasets and demonstrate that their system can accurately detect and associate faces from unconstrained videos and effectively learn robust and discriminative features for recognition. Huo et al. [19] tackle n-shot face recognition in videos using metric learning methods and similarity ranking models, comparing a Siamese network with contrastive loss to a Triplet Network with triplet loss. They show that feature representations learned with triplet loss are significantly better in their setting, and that learning spatio-temporal features from video sequences is beneficial for face recognition in videos. Rivero et al. [24] propose an adaptive aggregation scheme based on ordered weighted average (OWA) operators, and develop two different implementations to validate its suitability for image-to-video face recognition. Nevertheless, the current state of spatio-temporal face recognition research is insufficient, as the problem of face recognition in forensic videos is still open, and the results are not generalizable to real-world scenarios.

To avoid processing a whole video, keyframe extraction methods for face recognition in videos have been developed. Abed et al. [5] propose a method based on face quality and deep learning. The first step is face detection using the MTCNN detector, which detects five landmarks (the eyes, the two corners of the mouth, and the nose) and then limits face boundaries to a bounding box and from there provides a confidence score. This method involves two steps: the generation of face quality scores using three face feature extractors (Gabor, LBP, and HoG), and the training of a deep Convolutional Neural Network to select frames with the best face quality. Bahroun et al. [7] propose a keyframe extraction method based on face image quality for video surveillance systems. Data is reduced by rejecting frames without faces, and then face images are clustered by identity. A set of candidate frames is then selected, and the face quality assessment is based on four metrics (pose, sharpness, brightness, and resolution). The frame with the best face quality is considered a keyframe. Experimental tests were conducted on several datasets to demonstrate the effectiveness of the proposed method compared to other state-of-the-art approaches. The issue with some existing methods of face image quality computation is their dependence on subjective or indirect measures of quality, which may not necessarily align with the needs of face recognition systems. In contrast, these newer methods, as exemplified by the works of Abed et al. [5] and Bahroun et al. [7], provide a more direct measure of face image quality, which is closely tied to the performance of the face recognition model itself.

Face image quality assessment for improving face recognition in videos has also been considered. Terhorst et al. [9] propose the SER-FIQ (Subjective and Objective Quality Factors of Images) method for assessing face image quality. They test the SER-FIQ method on the IJB-C [12] video dataset and show that it performs well in face recognition tasks. Meng et al. [10] propose the MagFace method, which uses a multi-attention guided face image quality assessment network. They test MagFace on the IJB-C videos and show it outperforms other state-of-the-art methods. Ou et al. [11] propose the SDD-FIQA method, which uses a single shot detector to evaluate face image quality. They test SDD-FIQA on the IJB-C videos and show that it performs well in face recognition tasks. However, these works only evaluate face image quality in 1:1 (face verification) image-to-video scenarios, and do not consider the use of temporal information for face recognition as they use the frames as if they were isolated images.

Blind face restoration, which refers to the task of restoring faces in images without knowledge of the specific degradation processes they underwent, presents a complex challenge due to the inherent uncertainty stemming from its ill-posed nature and the potential loss of crucial details in degraded inputs. Together with super resolution techniques, they are emerging as important tools in improving image quality for various tasks, including face recognition. In a recent study, a novel approach has been proposed to handle the problem of blind face restoration, which is typically a highly ill-posed problem. Zhou et al. [20] introduces a learned discrete codebook prior in a small proxy space, reducing the uncertainty and ambiguity of restoration mapping by casting the process as a code prediction task. The approach is called CodeFormer, a Transformer-based prediction network that models the global composition and context of low-quality faces for code prediction. This technique enables the discovery of natural faces closely approximating the target faces, even with severely degraded inputs. The study showed that CodeFormer outperforms state-of-the-art methods in both quality and fidelity, exhibiting superior robustness to degradation. The results were validated on both synthetic and real-world datasets, further underscoring the effectiveness of the method in addressing the challenges of face restoration and super resolution. Despite these advancements, the use of such advanced preprocessing techniques for image-to-video face recognition, particularly in the context of forensic investigations, is still an open research question.

## 3 Methodology

**Fig. 3:**
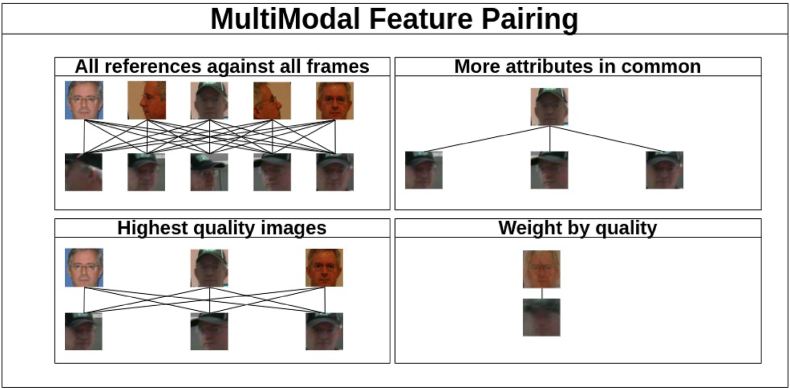
Examples of multimodal feature-pairing.

We propose a systematic workflow, illustrated in Fig. 2, that is segmented into various interconnected stages. The process commences with the curation of ‘Images-to-Video scenarios‘ incorporating both reference images and surveillance videos. These inputs undergo a ‘Multimodal feature pairing’ stage, further detailed in Fig. 3. In this stage, all frames are compared to one another; frames of the highest quality are paired, as are frames with shared attributes between the reference images and the video. Additionally, a frame weighted by quality from the reference images is paired with a similarly weighted frame from the video. After generating a biometric score *s*, the workflow advances to the ‘Calibration’ phase. During this stage, scores are calibrated using distribution models derived from both within-source variability (WSV) and between-source variability (BSV). This calibration is done with data from an ‘Images Database.’ Subsequently, the calibrated scores are transformed into a ‘Likelihood Ratio (LR),’ which is then subjected to a ‘Validation’ phase. During validation, external data from ‘ENFSI tests‘ involving the LR estimations of 18 participants, is incorporated. This offers a robust evaluation mechanism for the computed LRs, utilizing the ‘Cost-Log Likelihood Ratio !!insert-eqn13/!¡ as a metric to evaluate the strength of the evidence [14].

To estimate the LR as a measure of the strength of the evidence, the LR, we express it as the Score based Likelihood Ratio (SLR), defined as:

(1) $$SLR\left(s\right)=\frac{P\left(s|H_{p},I\right)}{P\left(s|H_{d},I\right)},$$

where *s* is the biometric score, *H*_*p*_ is the null hypothesis that the evidence originates from the same source, and *H*_*d*_ is the alternative hypothesis that it comes from a different source. Logistic Regression is employed to fit the probability functions *P*(*s*|*H*_*p*_, *I*) and *P*(*s*|*H*_*d*_, *I*), considering the background information available in the case.

It is crucial to recognize that the assumptions underlying the calculation of SLRs can significantly impact their values and interpretations, as demonstrated in the diverse forensic disciplines covered by Hepler et al. [25] and Ommen & Saunders [26]. Hepler et al. [25] underscore the variability of SLRs under different, yet plausible, assumptions, emphasizing the need for transparent and explicit model definitions. Ommen & Saunders [26] further elaborate on the complexities of source-level identification in forensic evidence, distinguishing between common and specific source problems. This distinction is critical for accurate and reliable application of SLRs, as it affects the choice of statistical models and the interpretation of evidence in both investigative and judicial contexts. Therefore, in developing SLRs for biometric systems, a comprehensive understanding of these frameworks and their implications is essential for reproducible research and for guiding future improvements in the field.

The workflow involves face detection, pairing of reference images and video frames, calibration of biometric scores using WSV and BSV, and validation against human performance. Following this methodology allows us to assess the likelihood of a person being present in a surveillance video, thus assisting in forensic investigations.

We propose an enhancement to the methodology by processing all frames in the video where a face is detected. For each of these frames, we compute the Face Image Quality (FIQ) and create an embedding vector *e*_*i*_, which represents the compressed representation of facial features. The FIQ scores are then used to apply a weighting scheme when combining the embedding vectors to form:

(2) $$e_{\mathrm{face}}=\overset{n}{\underset{i=1}{\sum }}q_{i}*e_{i},$$

This approach is applied to both the video frames and the reference images, allowing for a more comprehensive representation of the facial information. By incorporating FIQ-based weighting, we aim to improve the accuracy and reliability of face recognition in image-to-video comparisons.

The question we aim to answer is: How likely is the person as represented by a set of photos the same as the one appearing appearing in the surveillance video? Our focus is on the comparison of several reference images of the same person to a video in order to determine if the person appears in the video.

To estimate the likelihood ratio, the biometric score obtained from the comparison between the images and the video has to go through a process of calibration in which two distributions are computed: the WSV and the BSV. In this paper, we focus on two specific aspects of this process as it pertains to images-to-video comparisons: (1) methods for pairing reference images with videos, and (2) the use of different types of images, such as different qualities or different attributes, to create the WSV and BSV distributions during the calibration step. The biometric score must be calibrated using these distributions to estimate the likelihood ratio.

### 3.1 MultiModal feature pairing

In this work, we aim to improve the accuracy of likelihood ratio (LR) estimation in automated face recognition using images-to-video comparisons. Examples are shown in Fig. 3.

One approach is to employ score pairs derived from the shared attributes of the reference image and the video frame. Let *S*(*i*, *v*) denote the score for a given image *i* and video frame *v*. We define the score based on shared attributes:

(3) $$S\left(i,v\right)=\underset{a\in A}{\sum }\delta \left(a_{i},a_{v}\right)$$

where *A* is the set of all attributes, and *δ* is the Kronecker delta function. *δ*(*a*_*i*_, *a*_*v*_) is 1 if attribute *a* in image *i* matches attribute *a* in video *v*, and 0 otherwise.

Initially, we extract and calculate various attributes from all reference images and video frames, encompassing gender (for simplicity only comprising the categories man and woman), facial expression (including happy, angry, fear, and neutral), ethnicity (encompassing white, Asian, black, and Middle Eastern), yaw (representing frontal, slightly turned, and sideways orientations), pitch (Up, slightly up, frontal, slightly down, down), roll (frontal, slightly rolled, completely rolled), headgear, glasses, beard, and other occlusions (all of the latter booleans with value yes or no). Subsequently, we compare the attributes of each reference image with the attributes of every video frame, select pairs that exhibit the highest count of shared attributes, and we conduct likelihood ratio estimation as defined in equation (1).

By considering the attributes and iterating through various numbers of shared attributes, the algorithm can make more informed decisions, potentially enhancing the accuracy of the face recognition system. A summarized depiction of the algorithm can be found in algorithm 1.

Algorithm 1 Pair frames with most attributes in common

**Figure:**
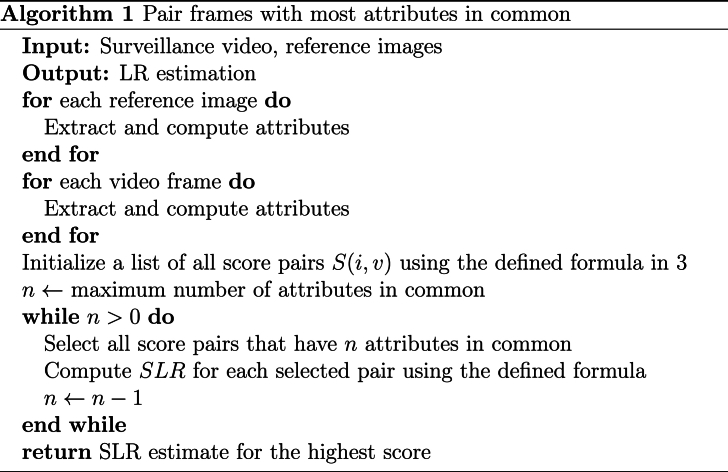

An alternative approach for performing the pairing is to match all the reference images with all the video frames, and then order them according to their quality. Once sorted by quality, the LR is calculated using all pairs. Subsequently, a process of pruning is applied, starting with the removal of 10% of the pairs with the lowest quality, followed by the removal of an additional 10% of the pairs, etc. The objective of this method is to determine if the information lost by discarding pairs is valuable, i.e. the SLR improves, which would indicate that the discarded images were noisy and thus detrimental to the face recognition system. An algorithm for this method is presented in algorithm 2.

Algorithm 2 Pair only the highest quality frames

**Figure:**
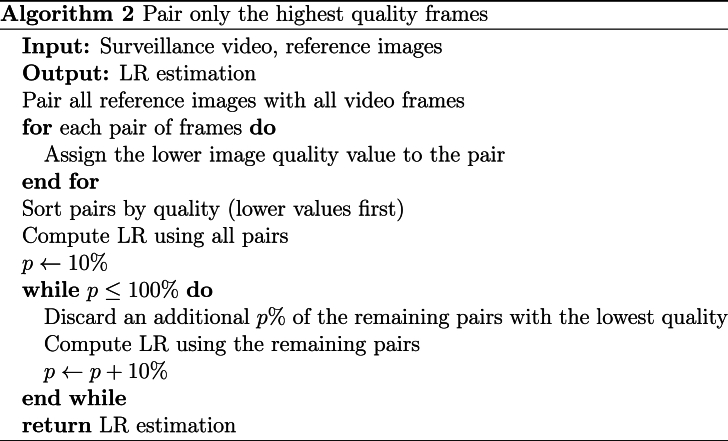

In addition, we propose to process all the frames in which a face is detected in the video, compute the FIQ of each frame, and create a combined embedding vector for the video using a weighting scheme based on the FIQ scores. Similarly, we process all the available reference images. This method is based on equation (2). This process is applied to both the video frames and the reference images. A summary of this process can be found in algorithm 3.

Algorithm 3 Weight all references and frames by quality

**Figure:**
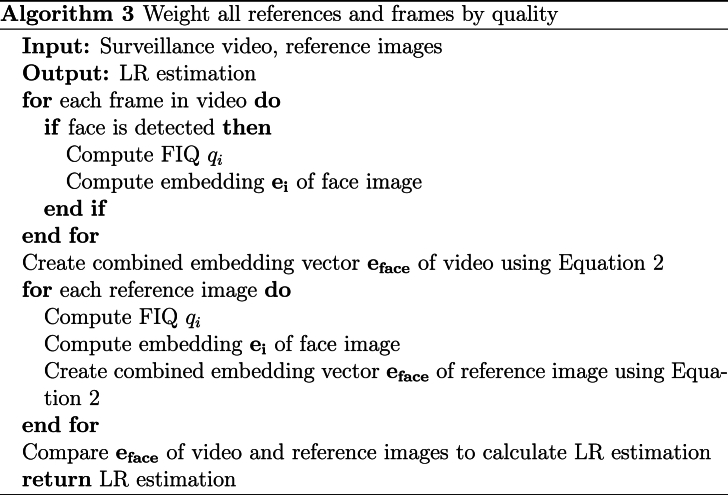

**Fig. 4:**
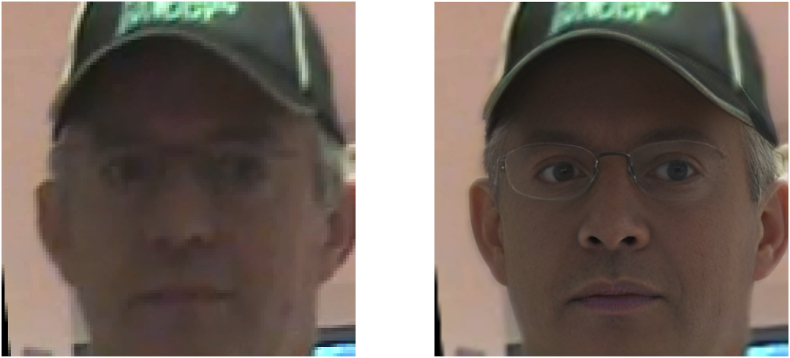
Example of super resolution image by Codeformer [20], on the left, the original, on the right, the processed image.

**Fig. 5:**
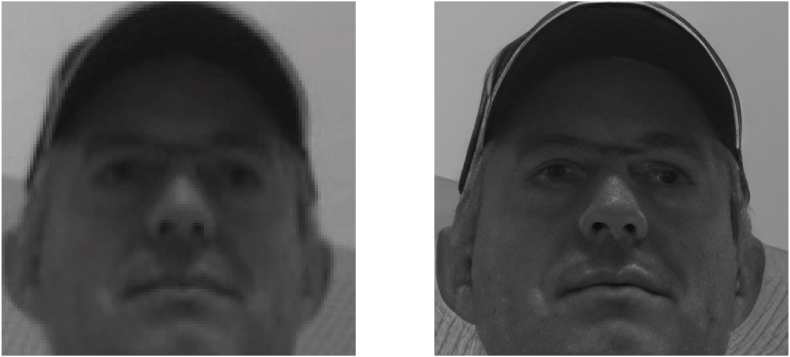
Example of super resolution image by Codeformer [20], on the left, the original, on the right, the processed image with artifacts on the eyes.

An alternate approach involves integrating the techniques used in algorithms 1, 3 and 2, while also adding an extra stage of preprocessing through the use of the super resolution CodeFormer. Our goal is to evaluate how such sophisticated image preprocessing might influence the accuracy and dependability of face recognition. See example of pre-processing in Fig. 4 and Fig. 5.

### 3.2 Calibration

To improve the accuracy of the LR estimation for automated face recognition in video, we will consider three different approaches for selecting images from the calibration database to use in the estimation process.

- **baseline** consists of using random images from the calibration database:
- **Same attributes:** Using images with the same attributes as the reference and video, such as pose or facial expression.
- **Quality pairs:** Using pairs that have the same FIQ group for the reference face and the combined face image qualities of the video frames. The FIQ group categorizes FIQ values into very low quality, low quality, medium quality, high quality, and very high quality.

By implementing these approaches, we aim to improve the accuracy of the LR estimation for automated face recognition in video.

## 4 Experiments

We will explore the workflow explained in section 3 doing experiments in the two parts of the method: pairing and calibration.

### 4.1 Datasets

Our study encompasses multiple datasets: ENFSI proficiency test [23], ForenFace [13], SCFace [27], and with respect to Ref. [17], we added the datasets XQLFW [21], and ChokePoint [22].

The ENFSI proficiency test 2015 focuses on matching mugshot images to CCTV video and includes 18 individual participants in 17 comparisons. ForenFace contains video sequences and extracted images of 97 subjects recorded with six different surveillance cameras. Its novelty lies in a subset of 435 images manually annotated, yielding forensically relevant annotation of almost 19,000 facial parts. SCFace has images taken in an uncontrolled indoor environment using five video surveillance cameras, consisting of 4160 static images and frames (in visible and infrared spectrum) of 130 subjects. The XQLFW dataset is a variant of the well-known Labeled Faces in the Wild (LFW) that focuses on cross-quality cases. It emphasizes the quality difference by containing only more realistically degraded images when necessary. It aids in assessing the robustness of face recognition models against various image quality challenges. ChokePoint was designed for real-world surveillance conditions. It was captured above several portals using an array of three cameras. The dataset features variations such as illumination, pose, sharpness, and misalignment. It comprises 48 video sequences and 64,204 face images of 54 subjects.

All these datasets consist of video sequences and face images with variations in illumination, pose, and sharpness. The study's objective is to train and test the performance of the proposed method on these datasets, seeking the most effective method to enhance the accuracy of the LR estimation for automated face recognition in video. A summary of these datasets can be found in Table 1.

**Table 1:** Summary of the five datasets.

Type	Dataset	Subjects/Images	Cameras	Description
Calibration	ForenFace	97/ $\tilde{4}$ 000	6	Forensic annotations
				Video & images
	SCFace	130/4160	5	Indoor images
				Static imgs & frames
	XQLFW	3743/7263	N/A	LFW [4] variant
				Emphasizes FIQ
				Degraded images
	ChokePoint	54/64,204	3	Dif. Pose & illumination
				Video sequences
Test	ENFSI	18/NA	N/A	Mugshot and CCTV
				Individual comparisons

### 4.2 Face recognition models and face quality models

We chose to use three face recognition models, ArcFace, Facenet, and QMagFace [28], in our experiments, because they all have been proposed recently, have demonstrated state-of-the-art performance, and all have different characteristics. ArcFace has a clear geometric interpretation and significantly enhances the discriminative power. Facenet directly learns a mapping from face images to a compact Euclidean space where distances correspond to a measure of face similarity, which makes it highly generalizable. QMagFace combines a quality-aware comparison score with a recognition model based on a magnitude-aware angular margin loss, making it suitable to enhance the recognition performance under unconstrained circumstances. We implemented ArcFace and Facenet from Ref. [29].

We use two quality models, SER-FIQ [9] and SDD-FIQA [11], as they both are unsupervised methods that have been shown to outperform state-of-the-art approaches in face image quality assessment and have good generalization across different recognition systems. SER-FIQ is based on the robustness against dropout variations as a quality indicator, and avoids the training phase completely. SDD-FIQA generates quality pseudo-labels by calculating the Wasserstein Distance (WD) between the intra-class and inter-class similarity distributions, which has been demonstrated to surpass state-of-the-art methods by an impressive margin.

### 4.3 Experimental cases

- **Experiment 1: Highest Number of Common Attributes.** Explained in algorithm 1. We aim to assess if using pairs that share attributes (multi-attribute, e.g., pairs with the same pose or facial expression) outperforms pairs that have nothing in common. We perform the LR estimation with 10,000 random images from the calibration set and 0 to 6 attributes in common (pitch, yaw, roll, facial expression, age, and gender).
- **Experiment 2: Quality-Based Drop.** Explained in algorithm 2. We aim to assess the influence of using the highest-quality frames on the LR estimation. We perform the experiment using 10,000 random images from the calibration dataset and compute the LR estimation by dropping 10% of the poorest quality face images in each iteration. We use the ENFSI test 2015 dataset for this experiment.
- **Experiment 3: Weighted Face Quality Images.** Explained in algorithm 3. We aim to assess the effect of weighting frames by quality on the LR estimation. We use 10,000 random images from the calibration dataset for this experiment.
- **Experiment 4: Calibration.** Explained further in the text. Once the comparison of images-to-video is computed, we aim to assess the difference in calibration using random images or images with the same FIQ as the test pair.
- **Experiment 5: Super Resolution Preprocessing.** In this experiment, we incorporate all the methods from Experiments 1–4 but with an additional layer of preprocessing using super resolution CodeFormer [20]. We aim to assess the impact of advanced image preprocessing on the face recognition accuracy and reliability.

### 4.4 Validation

To assess the performance of our proposed methods, we have selected the log-likelihood ratio cost (*C*_*llr*_) as the primary metric. This decision was influenced by the dual ability of *C*_*llr*_ to effectively represent both discrimination (the system's capacity to distinguish between individuals) and calibration (the accuracy of probabilistic estimates in decision making), which are critical in forensic face recognition systems [14]. Additionally, considering the extensive range of attributes and quality factors addressed in our study, using Tippett or Empirical Cross-Entropy (ECE) plots would have introduced significant complexity. These approaches, while informative, tend to generate a vast amount of data that can be challenging to analyze and compare, especially when dealing with a multitude of variables like attributes and quality levels. *C*_*llr*_ is defined as:

(4) $$C_{\mathrm{llr}}=\frac{1}{2N_{p}}\underset{i_{p}}{\sum }\log_{2}\left(1+\frac{1}{SLR_{i_{p}}}\right)+\frac{1}{2N_{d}}\underset{j_{d}}{\sum }\log_{2}\left(1+SLR_{j_{d}}\right),$$

where the indices *i*_*p*_ and *j*_*d*_ respectively denote summing over the computed SLR scores using equation (1) for each face pair comparison. Specifically, *i*_*p*_ sums over cases where the proposition for the prosecutor is true, while *j*_*d*_ sums over cases where the proposition for the defense is true. The variable *N* refers to the number of samples for each proposition. Minimizing the value of *C*_*llr*_ implies an improvement of both discrimination and calibration performance of the automated system [14]. The value ranges from zero (perfect decision making), to infinity (completely wrong). A value of one indicates the system makes a random selection. A value larger than one indicates the system is making a decision worse than random, i.e. supporting the prosecution hypothesis when it should support the defense hypothesis or vice versa.

In addition, we use boxplots to assess the impact of discarding pairs on the variability of our results, for both *i*_*p*_ (the summands corresponding to the prosecutor's proposition) and *j*_*d*_ (the summands corresponding to the defense's proposition). Specifically, we plot boxplots on the *C*_*llr*_ metric for each quality drop, indicating the percentiles of 25, median, and 75. The use of boxplots allows us to visualize the distribution of the *C*_*llr*_ metric and better understand how discarding pairs impacts the variability of the results, measure our approach for validating the performance of our proposed methods, and assess the impact of discarding pairs on the variability of the results.

## 5 Results

This section presents the findings of our investigation. The first subsection focuses on the correlation between facial image quality and various attributes such as gender, pose, race, and facial expression. The second subsection delves into the results of several experiments aimed at understanding the effect of different pairing methods on SLR estimation.

### 5.1 Correlation between facial image quality and attributes

**Fig. 6:**
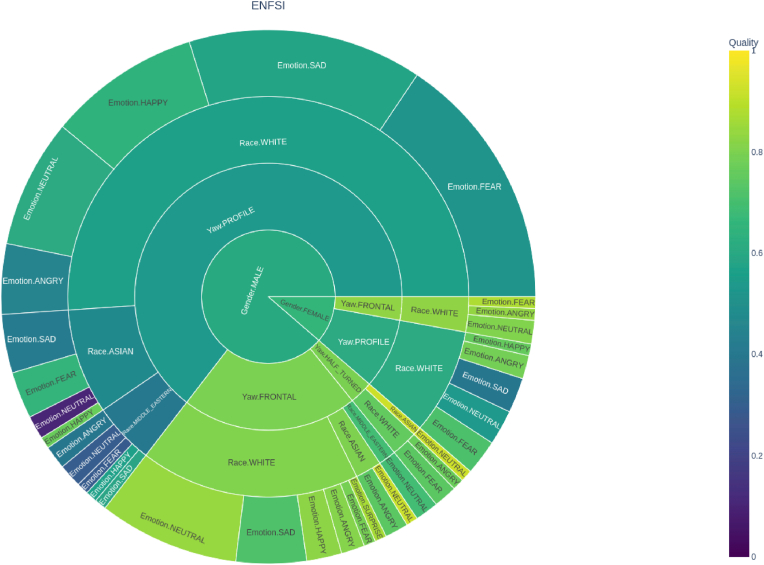
Facial attributes in the ENFSI database according to quality model SER-FIQ [9].

**Fig. A.11:**
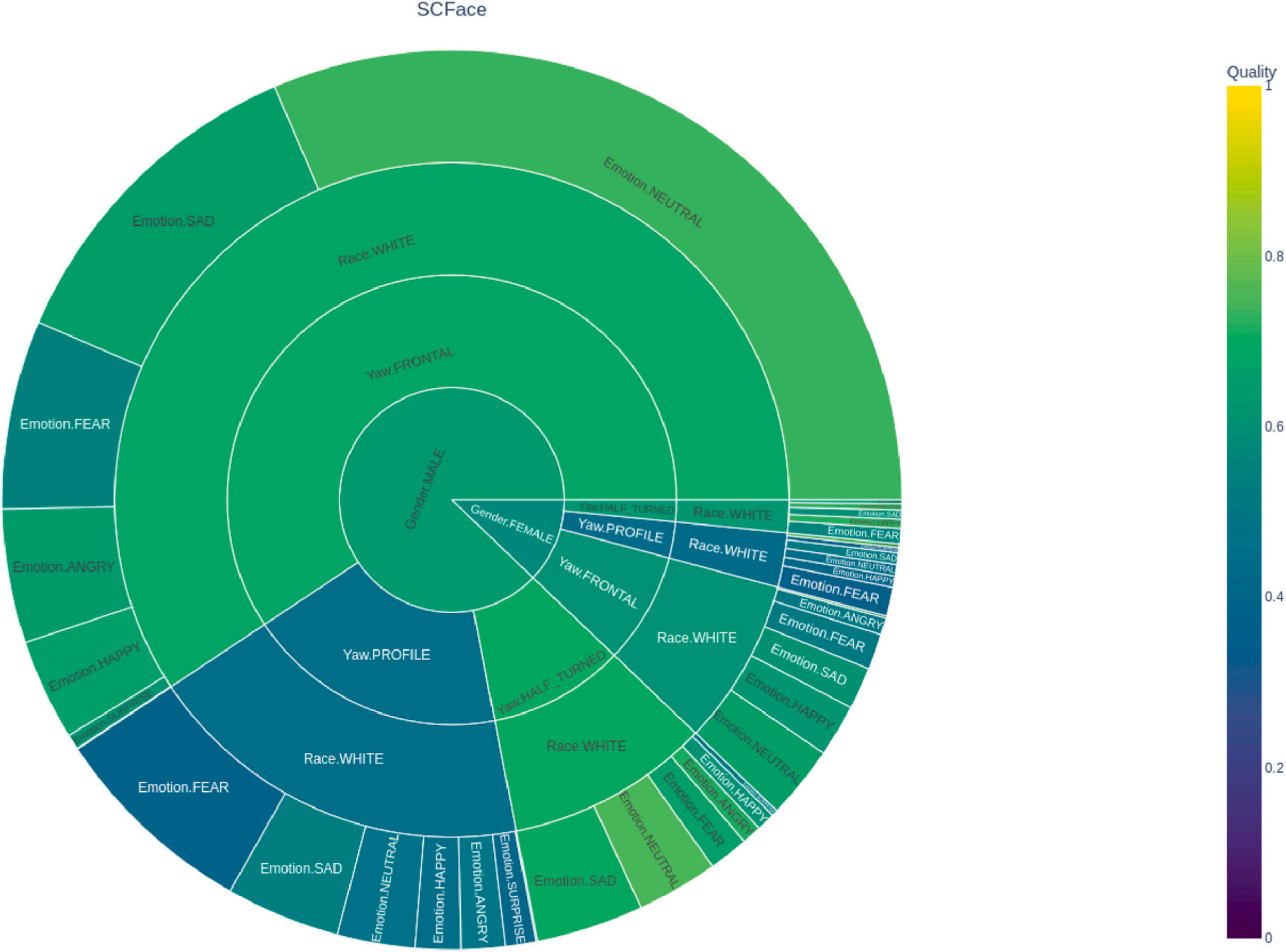
Facial attributes in the SCFace database according to quality model SER-FIQ [9].

**Fig. A.12:**
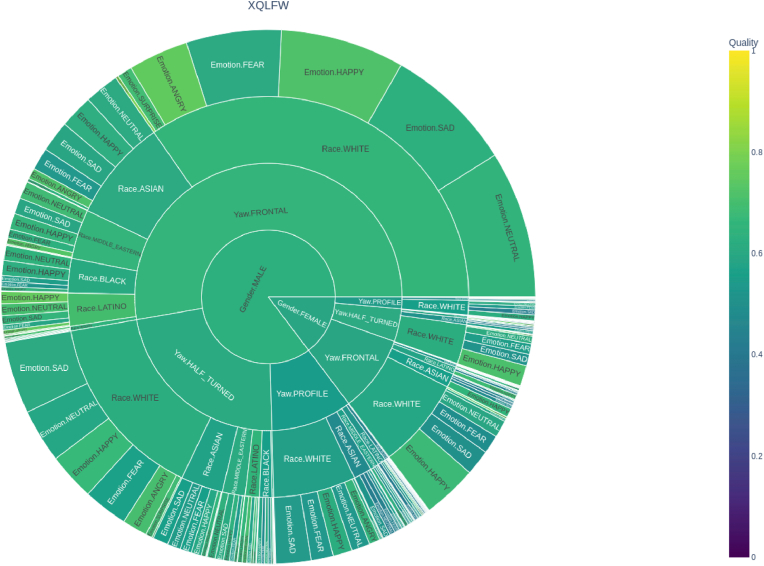
Facial attributes in the XQLFW database according to quality model SER-FIQ [9].

**Fig. A.13:**
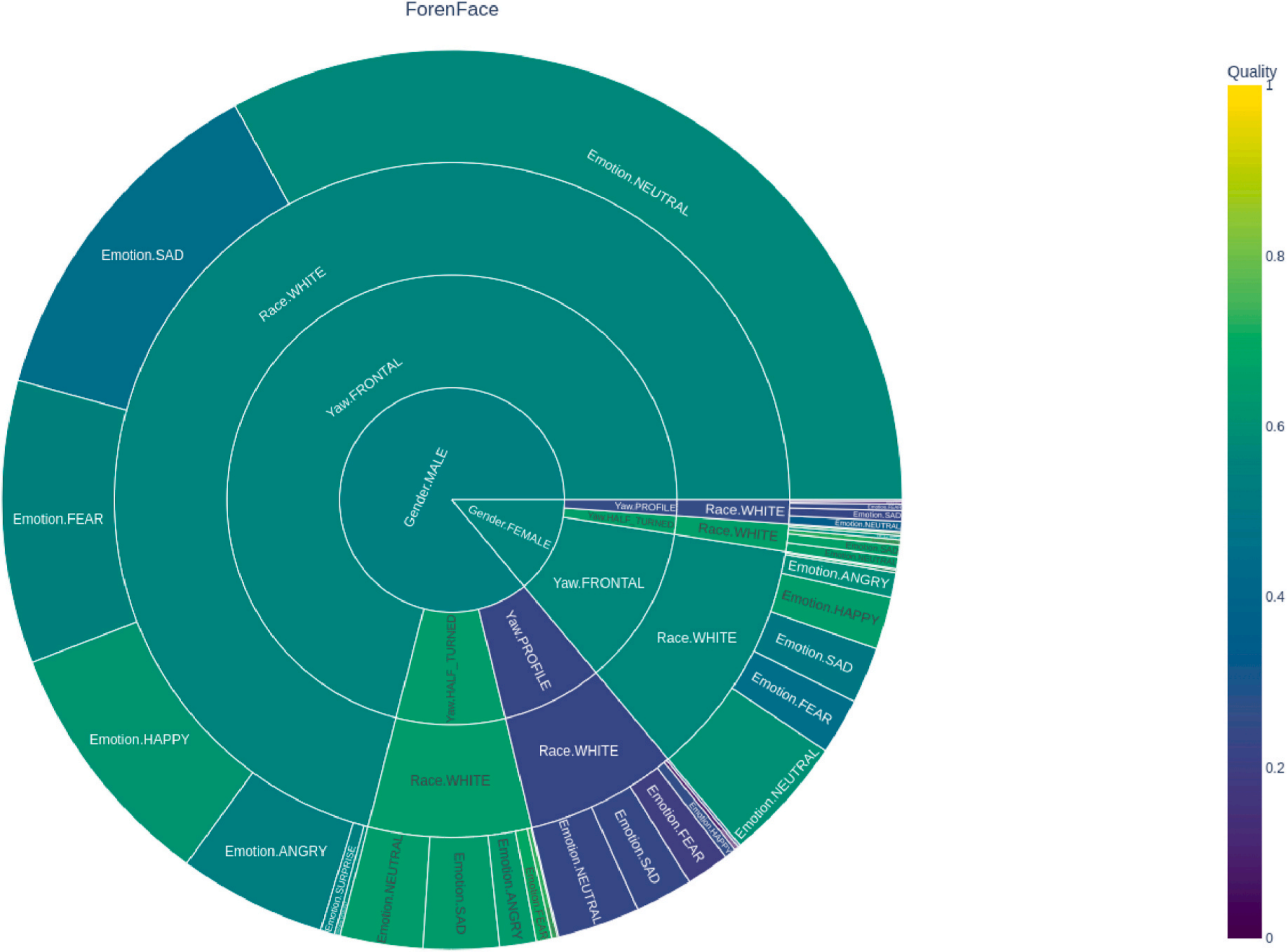
Facial attributes in the ForenFace database according to quality model SER-FIQ [9].

**Fig. A.14:**
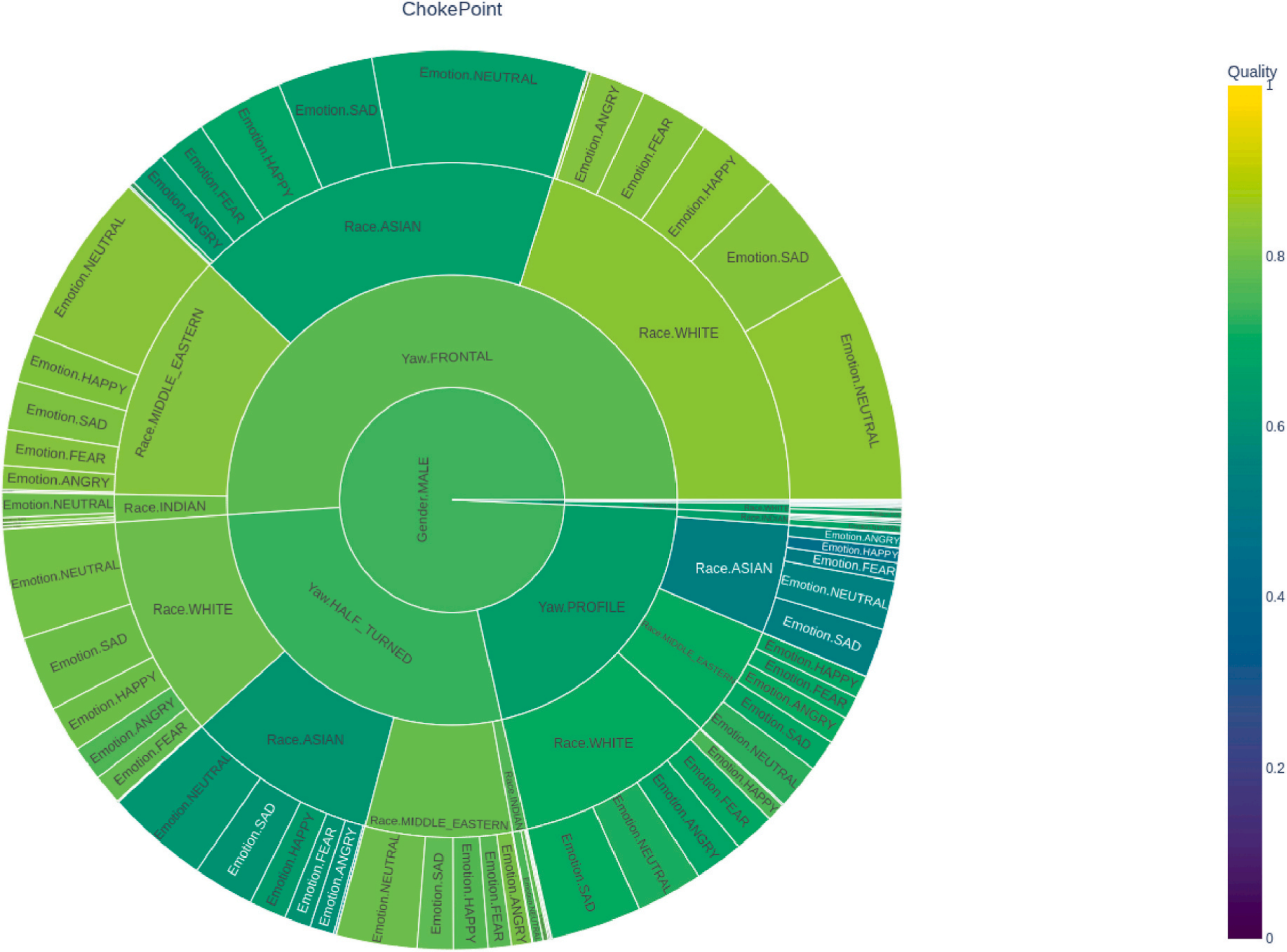
Facial attributes in the ChokePoint database according to quality model SER-FIQ [9].

To investigate the correlation between facial image quality and various facial attributes, Fig. 6 presents the results of a study conducted on the test dataset ENFSI 2015. These facial attributes include gender, pose (specifically yaw), race, and facial expression. The results for the calibration datasets—SCFace, XQLFW, ForenFace, and ChokePoint—are presented in Figures A11, A12, A13, and A14, respectively, which can be found in the appendix section.

To visually depict the intricate relationship between these attributes and the resulting image quality, we chose to use a sunburst hierarchical graph. This type of graph offers a compact and intuitive representation of hierarchical data across multiple dimensions. The order of attributes in the sunburst graph was chosen strategically to reflect their relevance and potential interactions. Gender, as the first attribute, is an important factor in face recognition and analysis. Its inclusion allows us to examine if gender has a significant influence on the quality of facial images. Next, the attribute of yaw (pose) was selected to explore the impact of different face orientations on image quality. Pose plays a vital role as it can affect the visibility of facial features and details. Analyzing yaw within the sunburst graph enables us to discern how different pose angles relate to image quality. Ethnicity, as the third attribute, is crucial for understanding potential variations in image quality among different racial or ethnic groups. Its inclusion in the graph allows us to identify patterns or disparities that may exist in image quality based on ethnicity. Finally, facial expression was chosen as the last attribute in the sunburst graph. Facial expressions are essential for face recognition and emotional analysis. By including facial expression in the graph, we can assess whether different expressions impact the quality of facial images.

The sunburst diagrams depicted provide a clear visual representation of the relationship between these attributes and the resulting image quality. From the analysis, it is evident that pose plays a significant role in the quality of facial images. Particularly, in terms of recognition, profile poses are associated with lower quality images, indicating a potential challenge in capturing sufficient detail and features in such poses.

Interestingly, gender does not appear to significantly influence the quality of facial images, suggesting that both male and female faces can be captured with comparable quality under the same conditions.

In terms of race, a noticeable pattern is seen in the XQLFW dataset, where images of individuals of Caucasian ethnicity seem to exhibit higher quality compared to other races. This could be due to many factors, such as lighting conditions, camera characteristics, or image processing techniques, and warrants further investigation.

**Fig. 7:**
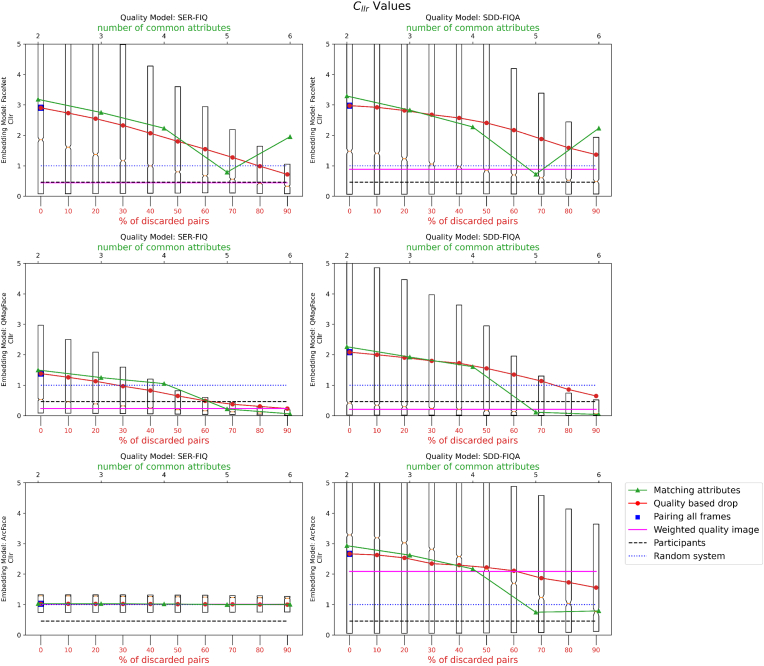
Graphical representation of the *C*_*llr*_ (log-likelihood ratio cost) values after calibration with attributes: yaw (top), pitch (middle), roll (bottom).

Results of four experiments on the effect of using different pairing methods on LR estimation in face recognition in videos are presented in Fig. 7. The experiments demonstrate how the quality of evidence and the number of common attributes influence the LR. A high LR indicates a match (same person) decision, and a low LR indicates non-match (different person). The quality assessment of the LR is based on the ground truth and is examined through the transformation into log_2_(1 + LR) for low LRs and $\log_{2}\left(1+\frac{1}{\text{LR}}\right)$ for high LRs. These transformations normalize the LR values, allowing for a more comparative analysis.

The calibrated log-likelihood ratio cost (*C*_*llr*_) presented in the graphs is indicative of the evidence's discriminative power. It is observed that as the quality of the attributes improves, or as the number of common attributes increases, both the *C*_*llr*_ value and its dispersion decrease, suggesting more reliable and consistent LR estimates.

**Fig. 8:**
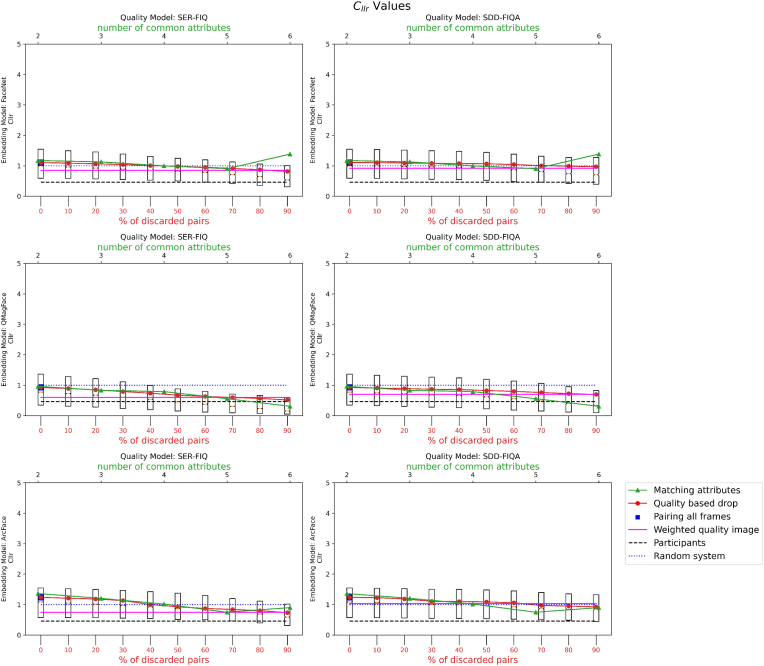
Graphical representation of the *C*_*llr*_ (log-likelihood ratio cost) values after calibration of random images.

**Fig. 9:**
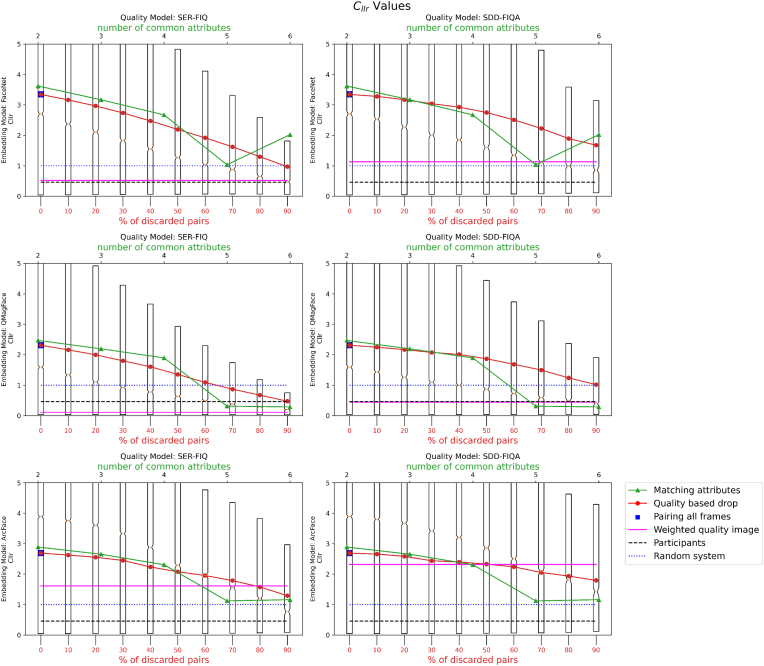
Graphical representation of the *C*_*llr*_ (log-likelihood ratio cost) values after calibration with quality filter.

Results corresponding to no filters and filters according to quality are presented in Fig. 8, Fig. 9 respectively.

**Fig. 10:**
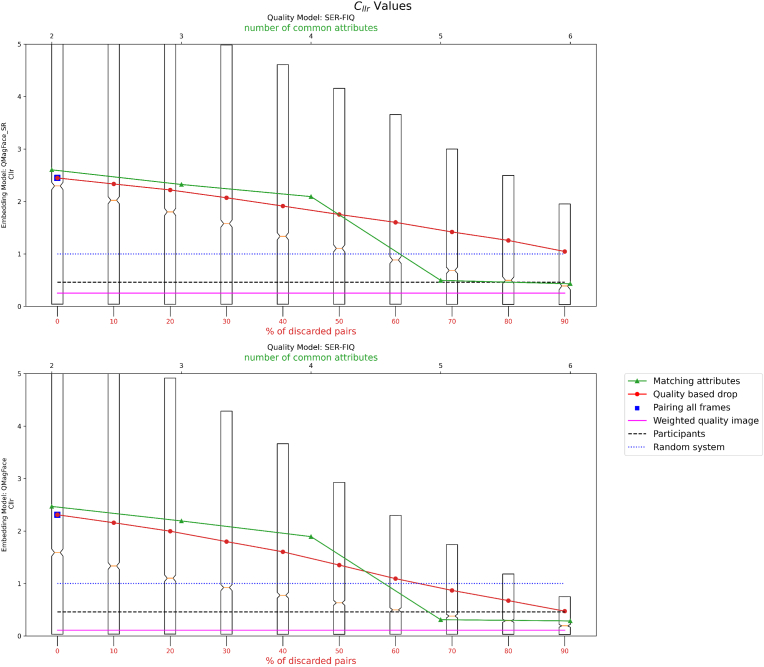
Graphical representation of the *C*_*llr*_ (log-likelihood ratio cost) values after the application of super-resolution processing (top). In the bottom graph, *C*_*llr*_ without pre-processing.

The outcome of applying super resolution is represented in Fig. 10. The former diagram indicates an enhancement in the quality of face images as per the Face Image Quality (FIQ) metric following the application of the super-resolution algorithm. Conversely, the latter figure suggests the results deteriorate after the super-resolution processing.

To further analyze LR results, as shown in Table 2, which created using data from Fig. 10) with the non pre-processed data. The first column of the table represents the percentage of images selected for analysis, not those discarded. Therefore, 10% in the table corresponds to 90% in the graph. It is observed that the LR (and LLR) values for y = 0 (different images) do not vary significantly, remaining around 0.3. However, there is a notable improvement in the LR when only the best pairs of images are selected. In addition, a decrease in data dispersion is noted. Apart from the standard deviation, the *σ*/average ratio has been included in the table, allowing for a more effective comparison between y = 1 and y = 0 cases. This demonstrates that the pairs of same images (y = 1) experience substantial improvement, while the pairs of different images (y = 0) show minimal improvement when reducing the number of pairs analyzed.

**Table 2:** Analysis of image pairs based on LR and LLR values.

**Best Quality Pairs**	**match/non-match**	**Average(LRs predicted)**	***σ*LRs predicted**	***σ*/average**	**LLR**
10%	0	0.033	0.075	2.297	-1.485
	1	10.167	33.792	3.324	1.007
20%	0	0.036	0.077	2.115	-1.441
	1	6.674	25.534	3.826	0.824
30%	0	0.036	0.084	2.331	-1.443
	1	5.089	21.412	4.207	0.707
40%	0	0.033	0.075	2.263	-1.480
	1	4.032	18.717	4.642	0.606
50%	0	0.031	0.072	2.342	-1.515
	1	3.352	16.854	5.029	0.525
60%	0	0.031	0.072	2.341	-1.510
	1	2.817	15.436	5.479	0.450
70%	0	0.031	0.070	2.251	-1.509
	1	2.472	14.352	5.805	0.393
80%	0	0.033	0.084	2.566	-1.485
	1	2.174	13.456	6.189	0.337
90%	0	0.034	0.090	2.658	-1.473
	1	1.936	12.705	6.564	0.287
100%	0	0.033	0.094	2.846	-1.481
	1	1.743	12.067	6.922	0.241

### 5.2 Effect of pairing methods on LR estimation

- **Results for Experiment 1.** The results indicate that having a higher number of attributes in common between the image pairs significantly lowers the *C*_*llr*_ value. This suggests that multi-attribute pairing may be an effective strategy for improving the accuracy of likelihood ratio (LR) estimation in biometric systems.
- **Results for Experiment 2.** Our findings reveal that using higher-quality frames leads to lower *C*_*llr*_ values, thereby enhancing the performance of the LR estimation. However, an interesting observation was that adding more frames does not uniformly improve *C*_*llr*_. In certain cases, incorporating lower-quality frames actually led to a worsened *C*_*llr*_, highlighting the importance of frame quality in the estimation process.
- **Results for Experiment 3.** When all frames were used but weighted by their quality, the *C*_*llr*_ values decreased, suggesting an improvement in the LR estimation. This finding implies that taking quality into account in a weighted manner can improve the system's overall performance, even when low-quality frames are included in the mix.
- **Results for Experiment 4.** We found variability in the calibration based on the set of images used. Using 20,000 images with the same attributes yielded a lower *C*_*llr*_ value compared to using 20,000 random images or images of the same quality as the test pair. This suggests that the choice of calibration set can have a substantial impact on the resulting *C*_*llr*_ and, by extension, on the performance of the LR estimation.
- **Results for Experiment 5.** Applying an advanced image preprocessing step through super resolution CodeFormer actually had a negative impact on the face recognition system's accuracy and reliability. The use of super resolution led to higher *C*_*llr*_ values, suggesting that it may not be beneficial for improving the performance of the likelihood ratio (LR) estimation in this specific context.

## 6 Discussion & conclusion

In our experiments, we found that using higher quality frames improves the performance of face recognition in video compared to using all frames. We explored different methods for pairing reference images with video frames. We found that using images with the same attributes as the reference and video, or similar FIQ score for the reference face and the combined face image qualities of the video frames, can improve the likelihood ratio estimation. Furthermore, we found that using a weighted quality average of all available reference and video frames improved results even more. On the other hand, slightly poorer results were obtained when pairing facial images based on the maximum number of common attributes. Although SDD-FIQA [11] outperforms SERFIQ in the LFW [4] and IJB-C [12] benchmarks, SERFIQ [9] seems more robust in our experiments. The *C*_*llr*_ obtained in the best case is close to 1, which is worse than the 0.45 of the expert participants in the ENFSI proficiency test [23]. This could be due to the difficulty and low face quality of the video frames used. Even discarding those with the poorest quality, the remaining ones are not suitable for the face recognition system in this experiment (ArcFace). However, using Facenet as the face recognition system in our experiments, we achieved a *C*_*llr*_ of 0.8, which is a better result. With QMagFace, we achieved even better results, with a *C*_*llr*_ of 0.26 using the method of the weighted quality image, surpassing the human participants in the ENFSI 2015 test, who scored a *C*_*llr*_ of 0.46. The best result was obtained by QMagFace and SER-FIQ with the method of pairing the highest number of attributes in common, with a *C*_*llr*_ of 0.13. The effectiveness of QMagFace was further underscored when paired with SER-FIQ based on the highest number of common attributes, where it reached an exceptional *C*_*llr*_ of 0.13.

Contrastingly, FaceNet and ArcFace demonstrated variable performance depending on the criteria used for pairing and frame selection. FaceNet exhibited enhanced behavior with an increasing number of common attributes, indicating its sensitivity to attribute matching. Meanwhile, ArcFace showed a preference for quality-based selection, as evidenced by its improved performance with quality discard and frames weighted by quality. This demonstrates the effectiveness of using FIQ as a metric to improve the performance of automated face recognition in video surveillance. The boxplots suggest there is less variability when more pairs are excluded.

It is worth noting that in surveillance settings, errors in attribute estimation can occur, which may affect the accuracy of face recognition systems that rely on shared attributes to select reference images and video frames. It is therefore crucial to investigate how errors in attribute estimation impact the performance of the proposed method, which pairs the highest number of attributes in common. It is also important to explore alternative approaches for selecting reference images and video frames that do not solely rely on shared attributes, such as deep metric learning, which can learn discriminative features for face recognition directly from the data. Future work should also consider examining the proposed methods on more diverse datasets, including those that present greater variability in facial attributes, to ensure the generalizability of the findings. Our results show the potential for using FIQ, spatio-temporal information and additional information, such as gait, clothes, or hair, to improve the performance of automated face recognition in video surveillance. Further research could explore the use of additional metrics for keyframe selection, and examine the performance of the proposed methods on a wider range of datasets and face recognition algorithms.

Intriguingly, our experiments also showed that preprocessing video frames with the super resolution Codeformer algorithm [20] did not lead to the anticipated improvement in face recognition performance. In fact, it seemingly deteriorated the outcome. One plausible explanation could be that the super-resolution process introduced some form of artifact or noise into the images that adversely affected the face recognition algorithms. Super-resolution algorithms like Codeformer generate high-frequency details that are not present in the original low-resolution image. If these details do not accurately represent the true high-resolution image, this could lead to mismatches compared with the reference face images, thereby deteriorating recognition performance.

In our study, we've delved into the intricacies of facial image quality metrics, attribute-based matching, and the impact of preprocessing techniques on face recognition. The importance of selecting high-quality frames has been reaffirmed, offering a tangible path forward for optimizing recognition performance in real-world scenarios. Our exploration into attribute-based pairings has illuminated both its potential benefits and areas requiring further study. In future work, it would be pertinent to explore the impact of ethnicity on face recognition systems, particularly considering concerns about potential ethnic bias. This exploration is crucial for enhancing transparency and fairness in these technologies, especially in law enforcement applications.

In conclusion, it is undeniable that facial image quality plays a pivotal role in face recognition, especially within video surveillance scenarios. Our results showcased the efficacy of using FIQ as a metric to enhance face recognition accuracy. Techniques such as utilizing weighted quality average and pairing based on shared attributes have proven to improve performance, underpinning the importance of considering these details in the recognition process. While not all explored methods yielded the expected enhancements—like the super resolution Codeformer algorithm, this exploration has provided valuable insights into the inherent complexities of automated face recognition, allowing us to better understand both its capabilities and limitations. These findings set a strong foundation for continuing advancements in the field, paving the way for further exploration of facial attributes, FIQ, and the potential integration of alternative super-resolution techniques.

## CRediT authorship contribution statement

**Andrea Macarulla Rodriguez:** Writing – original draft, Visualization, Validation, Software, Resources, Methodology, Investigation, Conceptualization. **Zeno Geradts:** Writing – review & editing, Supervision, Methodology, Funding acquisition, Conceptualization. **Marcel Worring:** Writing – review & editing, Supervision, Methodology, Conceptualization. **Luis Unzueta:** Writing – review & editing, Supervision, Conceptualization.

## Declaration of Generative AI and AI-assisted technologies in the writing process

During the preparation of this work the author(s) used GPT-4 in order to correct the English in the manuscript. After using this tool/service, the author(s) reviewed and edited the content as needed and take(s) full responsibility for the content of the publication.

## Declaration of competing interest

The authors declare that they have no known competing financial interests or personal relationships that could have appeared to influence the work reported in this paper.
